# Supplementary figures and images for: NF90 interacts with components of RISC and modulates association of Ago2 with mRNA
Source: BMC Biol. 2022 Sep 1;20:194. doi: 10.1186/s12915-022-01384-2 (PMC9438302; doi:10.1186/s12915-022-01384-2)

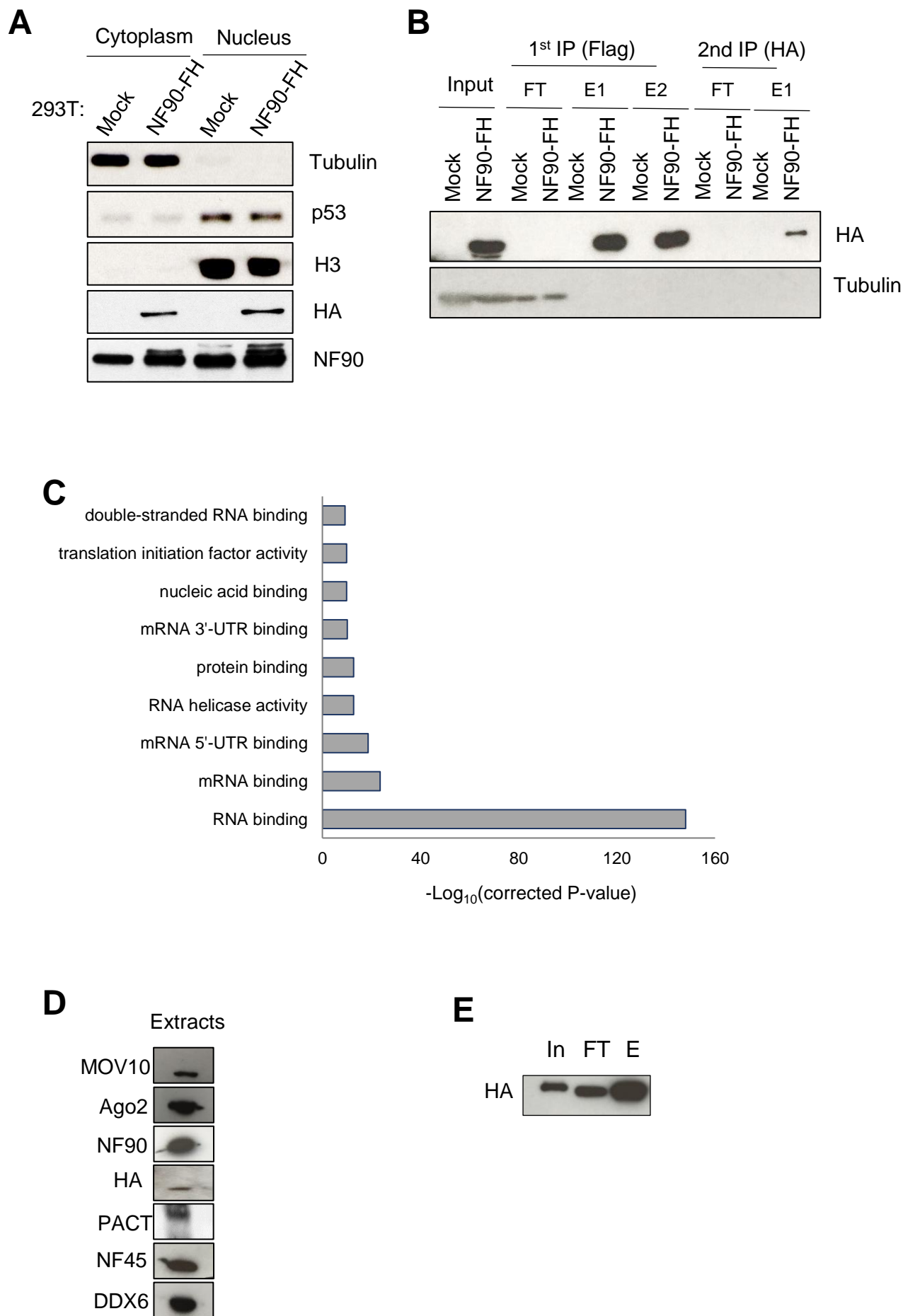

**Figure S1**

Supplement: Supplementary file 1 — Additional file 1: Fig. S1. NF90 interacts with proteins involved in translational repression and RNA processing. (A) Cytoplasmic and nuclear extracts of WT (mock) and NF90-FH stably overexpressing (NF90-FH) HEK293T cells were analyzed by Western blot using the indicated antibodies. (B) Cytoplasmic extracts described in A underwent tandem affinity purification using FLAG and HA antibodies. Samples were analyzed by western blot using the indicated antibodies (FT = flow through, E1 = elution 1; E2 = elution 2). (C) Molecular functions of NF90-associated proteins (n = 209; 3 or more peptides, FC>2) identified by mass spectrometry were analyzed using gene ontology. (D) Cytoplasmic extracts of NF90-FH overexpressing HEK293T cells used for FLAG immunoprecipitation followed by glycerol gradient sedimentations were analyzed by western blot using the antibodies indicated. (E) An aliquot of FLAG immunoprecipitate from NF90-FH overexpressing HEK293T cells used for glycerol gradient sedimentation were analyzed by western blot, using the antibodies indicated (In = input, FT = flowthrough, E = elution). [file 12915_2022_1384_MOESM1_ESM.pdf]

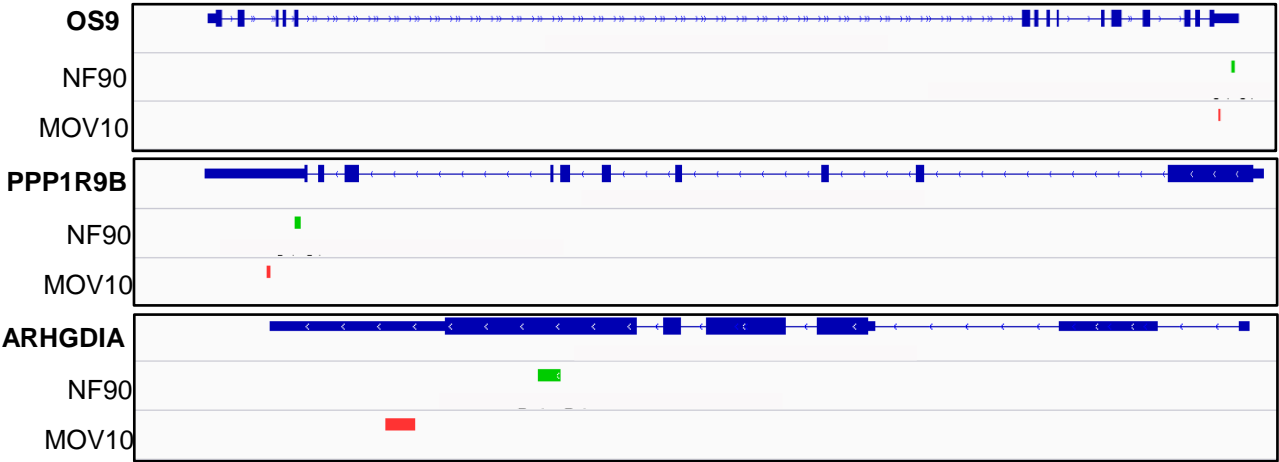

Figure S2

Supplement: Supplementary file 2 — Additional file 2: Fig. S2. NF90 and MOV10 can bind the same target mRNAs. Screenshots of NF90 and MOV10 eCLIP and iCLIP, respectively, showing regions associated with NF90 (green bars) and MOV10 (red bars) within selected target mRNAs. Introns are shown as thin lines, 5’ and 3’ UTRs are shown as medium lines and exons are shown as thick lines. The orientation of the transcript with respect to the genome is indicated by arrows. [file 12915_2022_1384_MOESM2_ESM.pdf]

**A**

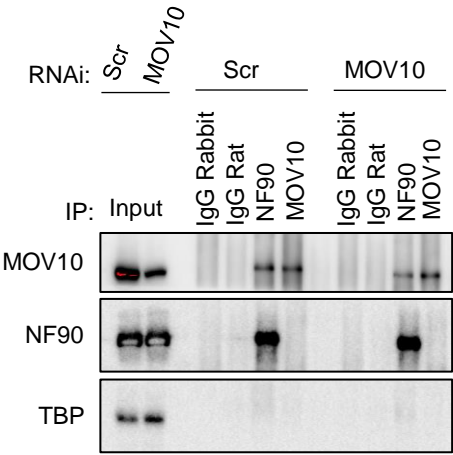

**B**

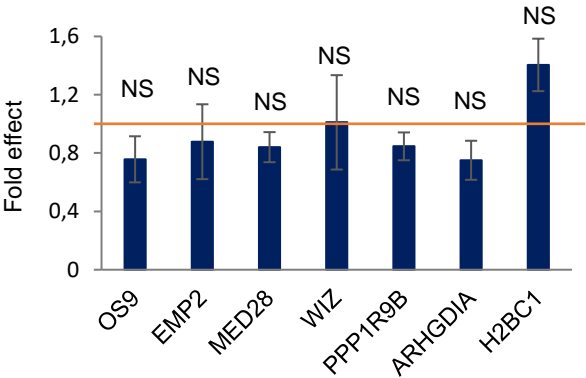

**Figure S3**

Supplement: Supplementary file 3 — Additional file 3: Fig. S3. MOV10 modulates NF90 association with common target mRNAs. (A) Extracts of HEK293T cells transfected with siRNAs targeting MOV10 or a nontargeting control (Scr) and immunoprecipitates obtained using antibodies anti-NF90, anti-MOV10 or control IgG were analyzed by Western blot using the indicated antibodies. (B) Total RNA obtained from HEK293T transfected with siRNAs targeting MOV10 or a non-targeting control (Scr) was analyzed by RT-qPCR using transcript specific PCR primers, as indicated. Values obtained in MOV10 knock-down condition were calculated relative to the control samples (siScr), which was attributed a value of 1 (red line). Data represent the mean ± SEM obtained from 4 independent experiments (NS indicates ‘Not Significant’, independent Student’s t test) (Additional file 9). [file 12915_2022_1384_MOESM3_ESM.pdf]

**A**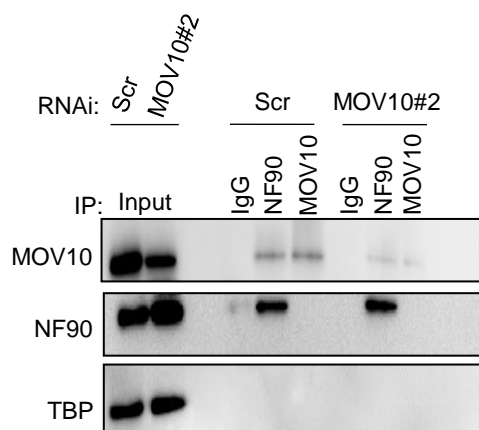**B**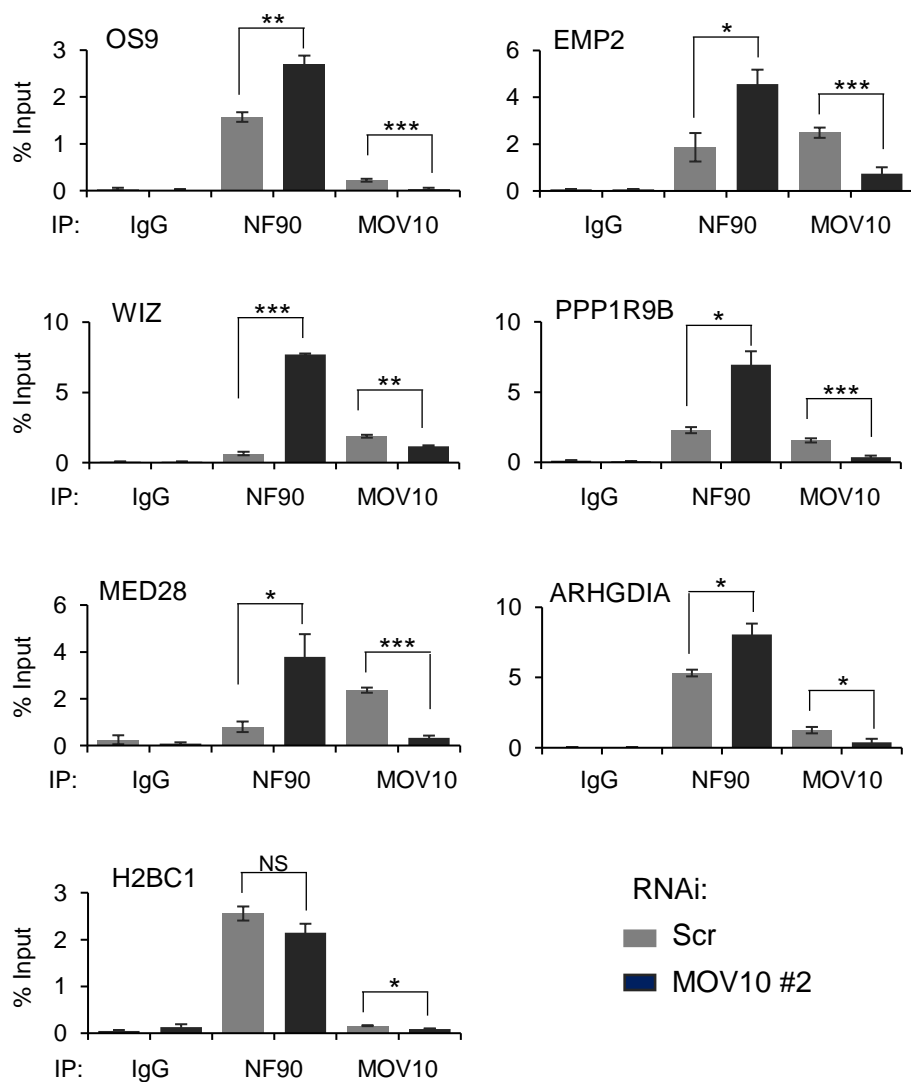**Figure S4**

Supplement: Supplementary file 4 — Additional file 4: Fig. S4. MOV10 modulates NF90 association with common target mRNAs. (A) Extracts of HEK293T cells transfected with siRNAs targeting MOV10 (MOV10#2) or a non-targeting control (Scr) and immunoprecipitates obtained using antibodies against NF90, MOV10 or control IgG were analyzed by Western blot using the indicated antibodies. (B) RIP analysis of HEK293T cells transfected with MOV10-targeting siRNA (MOV10 #2) or a non-targeting control (Scr), as indicated. RIPs were performed using anti-NF90, anti-MOV10 or a control IgG antibody, as indicated. An aliquot of input and Immunoprecipitates were analyzed using RT-qPCR using transcript-specific primers. Data represent mean ± SEM obtained from n > 6 independent experiments (*P < 0.05, **P < 0.01, ***P < 0.001, NS indicates Not Significant, independent Student’s t test). [file 12915_2022_1384_MOESM4_ESM.pdf]

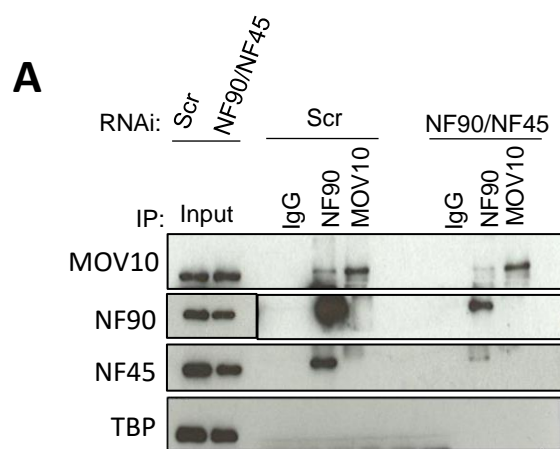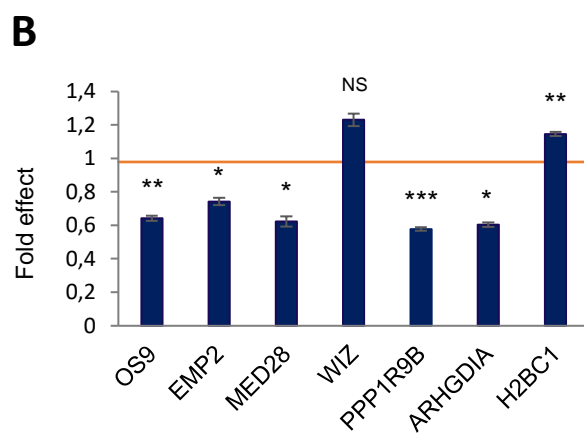

**Figure S5**

Supplement: Supplementary file 5 — Additional file 5: Fig. S5. NF90 modulates MOV10 association with common target mRNAs. (A) Extracts of HEK293T cells transfected with siRNAs targeting NF90 and NF45 or a non-targeting control (Scr) and immunoprecipitates obtained using antibodies anti-NF90, anti-MOV10 or control IgG were analyzed by Western blot using the indicated antibodies. (B) Total RNA obtained from HEK293T transfected with siRNAs targeting NF90 and NF45 or a non-targeting control (Scr) was analyzed by RT-qPCR using transcript-specific PCR primers, as indicated. Values obtained in NF90/NF45 knock-down condition were calculated relative to the control samples (siScr), which was attributed a value of 1 (red line). Data represent the mean ± SEM obtained from 4 independent experiments (*P < 0.05, **P < 0.01, ***P < 0.001, NS indicates Not Significant, independent Student’s t test) (Additional file 9). [file 12915_2022_1384_MOESM5_ESM.pdf]

**A**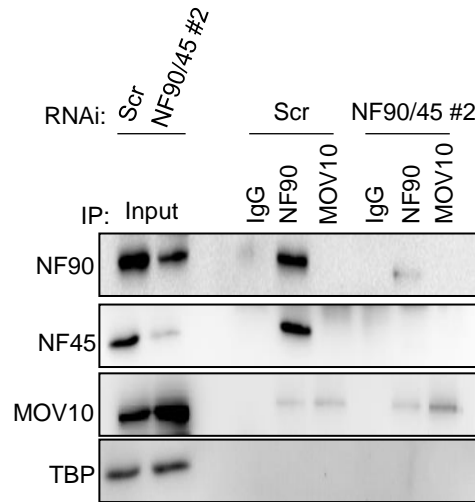**B**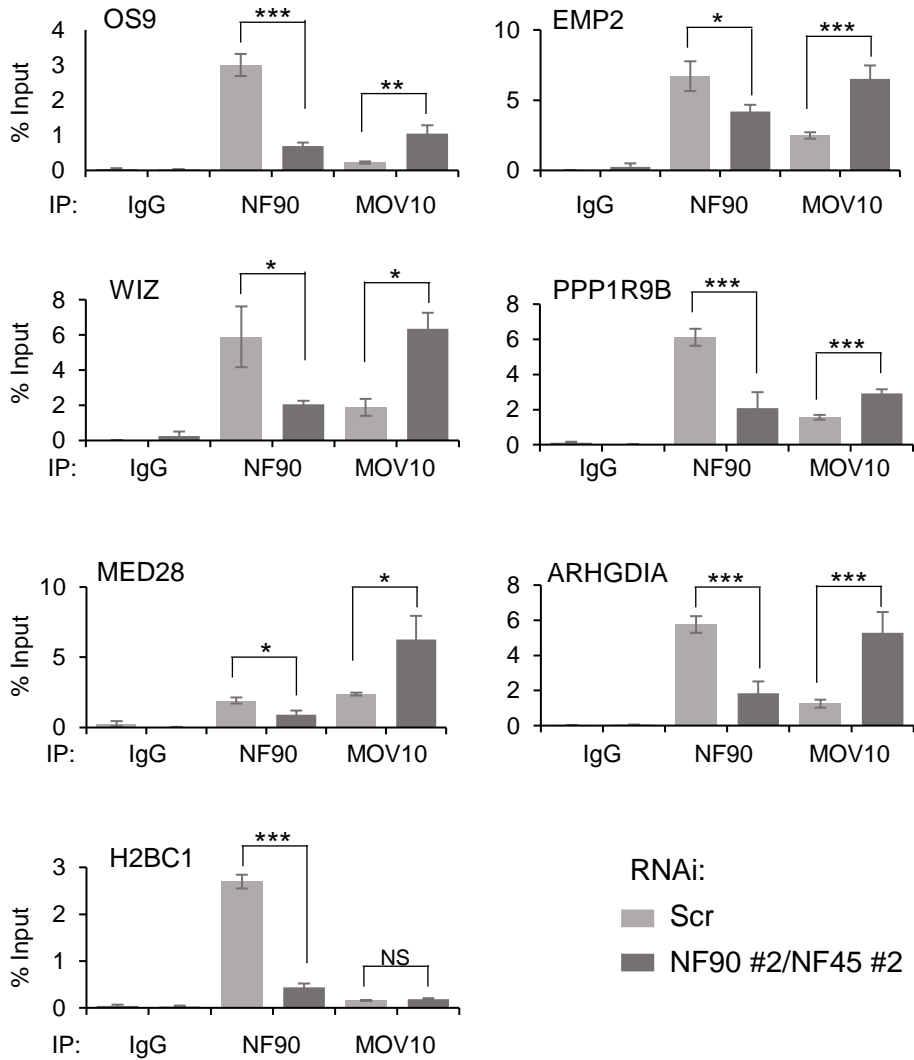**Figure S6**

Supplement: Supplementary file 6 — Additional file 6: Fig. S6. NF90 modulates MOV10 association with common target mRNAs. (A) Extracts of HEK293T cells transfected with siRNAs targeting NF90 and NF45 (siNF90#2/NF45#2) or a non-targeting control (Scr) and immunoprecipitates obtained using anti-NF90, anti-MOV10 or control IgG were analyzed by Western blot using the indicated antibodies. (B) RIP analysis of HEK293T cells transfected with NF90/NF45-targeting siRNAs (siNF90#2/NF45#2) or a non-targeting control (Scr), as indicated. RIPs were performed using anti-NF90, anti-MOV10 or a control IgG antibody. An aliquot of input and Immunoprecipitates were analyzed using RT-qPCR. ND indicates ‘Not Detected’. Data represent mean ± SEM obtained from n > 6 independent experiments (*P < 0.05, **P < 0.01, ***P < 0.001, NS indicates Not Significant, independent Student’s t test). [file 12915_2022_1384_MOESM6_ESM.pdf]

**A**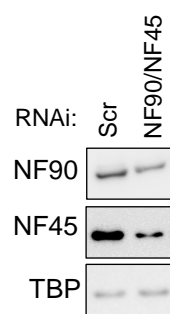**B**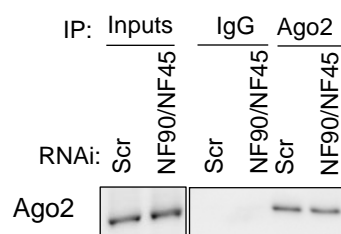**Figure S7**

Supplement: Supplementary file 7 — Additional file 7: Fig. S7. Downregulation of NF90/NF45 does not affect the expression of Ago2 (A) Extracts of HEK293T cells transfected with siRNAs targeting NF90/NF45 or a nontargeting control (Scr) were analyzed by Western blot using the indicated antibodies. (B) Immunoprecipitates obtained using anti-Ago2 or control IgG antibodies from extracts of HEK293T cells transfected with siRNAs targeting NF90/NF45 or a nontargeting control (Scr) were analyzed by Western blot using anti-Ago2 antibody. [file 12915_2022_1384_MOESM7_ESM.pdf]
