# Supplementary material for: NF90 interacts with components of RISC and modulates association of Ago2 with mRNA
Source: BMC Biol. 2022 Sep 1;20:194. doi: 10.1186/s12915-022-01384-2 (PMC9438302; doi:10.1186/s12915-022-01384-2)
Supplement: Supplementary file 8 — Additional file 8: Table S1. Double stranded siRNAs used in this study. Table S2. Primary antibodies used in this study. Table S3. Primers used in this study. Table S4. Proteins associated with NF90 in the cytoplasm, detected by tandem affinity purification followed by tandem mass spectrometry. Number of peptides refers to the number of unique peptides identified in the NF90 sample. Fold change indicates the number of peptides detected in the NF90 sample divided by the number of peptides detected in the mock control sample. ND in control indicates that no peptides were detected in the control sample. [file 12915_2022_1384_MOESM8_ESM.pdf]

Supplementary Table S1. Double stranded siRNAs used in this study.

siRNA	Sequence (5' to 3')
Scr	gcgcgcuuuguaggauucg(dTdT)
NF45	guggugauacucaagauucugccaa(dTdT)
NF45#2	agaaaaacuaauuaaagua(dTdT)
NF90	ccaaggaacucuaucacaa(dTdT)
NF90#2	ggaacucuaucacaauuugaaaaga(dTdT)
MOV10	guggaauuggaccgugucaagcuga(dTdT)
MOV10#2	ggucagauaucagcaaacaccacaa(dTdT)

Supplementary Table S2. Primary antibodies used in this study.

Antibody	Reference	Supplier
NF90	A303-651A	Bethyl Laboratories
NF45	A303-147A	Bethyl Laboratories
MOV10	A301-571A	Bethyl Laboratories
PACT	sc-81569	SCBT
HA	12CA5	Roche
DDX6	A300-461	Bethyl Laboratories
Ago2 WB	MABE253, clone 119A	Sigma-Aldrich
Ago2 RIP	03-110	Sigma-Aldrich
TUBULIN	DM1A clone, T6199	Sigma-Aldrich
TBP	sc-421	SCBT

Supplementary Table S3. Primers used in this study.

Primer	Forward (5' to 3')	Reverse (5' to 3')
OS9	CTGCCCTTAGTGATGTTTGGA	ATTCTACCATTTCCTCAGCAACA
EMP2	GACCCATCCACCATTCATTC	CCACTGTACCCAGCCAGTTT
MED28	GAGCAGGCAGTAGGATGAGG	CCCCTCCACAGGGATATTTT
WIZ	TTGGCTGCTCCTTCTTGTTT	GCCTGTTAATCCCTCCTTCC
PPP1R9B	GCCCTGCACTGATTTCTCAT	TATTTGGCACCTGGAAGAGG
ARHGDIA	TGCCTCTGCCTTTTCTGTCT	GCACTTGGTCCCTTGTTTGT
H2BC1	GTGCTAAAGCAGGTCCATCC	GCATGTTTAGCCAGCTCTCC
VEGFA	TGACAGGGAAGAGGAGGAGA	CGTCTGACCTGGGGTAGAGA

Supplementary Table S4. Proteins associated with NF90 in the cytoplasm, detected by tandem affinity purification followed by tandem mass spectrometry. Number of peptides refers to the number of unique peptides identified in the NF90 sample. Fold change indicates the number of peptides detected in the NF90 sample divided by the number of peptides detected in the mock control sample. ND in control indicates that no peptides were detected in the control sample.

Reference	Gene Symbol	Number of peptides	Fold Change
Q12906_ILF3_HUMAN	ILF3	40	ND in control
Q08211_DHX9_HUMAN	DHX9	29	2,42
Q9HCE1_MOV10_HUMAN	MOV10	25	ND in control
P11940_PABP1_HUMAN	PABPC1	25	2,78
O75533_SF3B1_HUMAN	SF3B1	22	3,67
Q7L2E3_DHX30_HUMAN	DHX30	21	ND in control
Q12905_ILF2_HUMAN	ILF2	21	ND in control
Q6PKG0_LARP1_HUMAN	LARP1	21	ND in control
Q9NZI8_IF2B1_HUMAN	IGF2BP1	21	3,00
Q15393_SF3B3_HUMAN	SF3B3	19	4,75
O60506_HNRPQ_HUMAN	SYNCRIP	18	ND in control
O43390_HNRPR_HUMAN	HNRNPR	17	ND in control
Q92900_RENT1_HUMAN	UPF1	17	ND in control
Q92841_DDX17_HUMAN	DDX17	17	2,13
P55884_EIF3B_HUMAN	EIF3B	17	2,13
P52272_HNRPM_HUMAN	HNRNPM	16	8,00
P98175_RBM10_HUMAN	RBM10	16	3,20
Q9Y6M1_IF2B2_HUMAN	IGF2BP2	15	ND in control
O43143_DHX15_HUMAN	DHX15	15	5,00
P19338_NUCL_HUMAN	NCL	15	5,00
P55265_DSRAD_HUMAN	ADAR	14	ND in control
P78347_GTF2I_HUMAN	GTF2I	13	ND in control
O00425_IF2B3_HUMAN	IGF2BP3	13	6,50
O75643_U520_HUMAN	SNRNP200	12	ND in control
P36578_RL4_HUMAN	RPL4	12	12,00
Q13310_PABP4_HUMAN	PABPC4	12	6,00
Q13435_SF3B2_HUMAN	SF3B2	12	6,00
P46781_RS9_HUMAN	RPS9	12	4,00
O15372_EIF3H_HUMAN	EIF3H	12	2,40
P39023_RL3_HUMAN	RPL3	11	ND in control
Q9NUL3_STAU2_HUMAN	STAU2	11	ND in control
P12956_XRCC6_HUMAN	XRCC6	11	5,50
P62701_RS4X_HUMAN	RPS4X	11	2,20
P55072_TERA_HUMAN	VCP	11	2,20
Q9NZB2_F120A_HUMAN	FAM120A	10	ND in control
P14866_HNRPL_HUMAN	HNRNPL	10	ND in control

Reference	Gene Symbol	Number of peptides	Fold Change
Q99873_ANM1_HUMAN	PRMT1	10	ND in control
P05455_LA_HUMAN	SSB	10	ND in control
O95793_STAU1_HUMAN	STAU1	10	ND in control
Q9NUD5_ZCHC3_HUMAN	ZCCHC3	10	ND in control
A5YKK6_CNOT1_HUMAN	CNOT1	10	5,00
Q9Y262_EIF3L_HUMAN	EIF3L	10	5,00
Q13347_EIF3I_HUMAN	EIF3I	10	3,33
Q14204_DYHC1_HUMAN	DYNC1H1	10	2,00
P62750_RL23A_HUMAN	RPL23A	9	ND in control
Q02878_RL6_HUMAN	RPL6	9	4,50
P62249_RS16_HUMAN	RPS16	9	4,50
P62269_RS18_HUMAN	RPS18	9	4,50
P08865_RSSA_HUMAN	RPSA	9	3,00
O15042_SR140_HUMAN	U2SURP	9	3,00
P67809_YBOX1_HUMAN	YBX1	9	3,00
P15880_RS2_HUMAN	RPS2	9	2,25
Q13813_SPTN1_HUMAN	SPTAN1	9	2,25
Q9NR30_DDX21_HUMAN	DDX21	8	ND in control
Q9H2U1_DHX36_HUMAN	DHX36	8	ND in control
Q9BVP2_GNL3_HUMAN	GNL3	8	ND in control
P48634_PRC2A_HUMAN	PRRC2A	8	ND in control
P46777_RL5_HUMAN	RPL5	8	ND in control
Q14157_UBP2L_HUMAN	UBAP2L	8	ND in control
P61221_ABCE1_HUMAN	ABCE1	8	8,00
Q9P2R3_ANFY1_HUMAN	ANKFY1	8	8,00
P60842_IF4A1_HUMAN	EIF4A1	8	8,00
Q15717_ELAV1_HUMAN	ELAVL1	8	8,00
P61313_RL15_HUMAN	RPL15	8	8,00
P62424_RL7A_HUMAN	RPL7A	8	8,00
P63244_GBLP_HUMAN	GNB2L1	8	4,00
P61247_RS3A_HUMAN	RPS3A	8	4,00
Q9H0D6_XRN2_HUMAN	XRN2	8	2,67
O75821_EIF3G_HUMAN	EIF3G	8	2,00
P39019_RS19_HUMAN	RPS19	8	2,00
Q15750_TAB1_HUMAN	TAB1	8	2,00
Q14692_BMS1_HUMAN	BMS1	7	ND in control
P26196_DDX6_HUMAN	DDX6	7	ND in control
P38919_IF4A3_HUMAN	EIF4A3	7	ND in control
P07910_HNRPC_HUMAN	HNRNPC	7	ND in control
P52292_IMA1_HUMAN	KPNA2	7	ND in control

Reference	Gene Symbol	Number of peptides	Fold Change
Q6P2Q9_PRP8_HUMAN	PRPF8	7	ND in control
P62829_RL23_HUMAN	RPL23	7	ND in control
P18124_RL7_HUMAN	RPL7	7	ND in control
P32969_RL9_HUMAN	RPL9	7	ND in control
Q9UQ35_SRRM2_HUMAN	SRRM2	7	ND in control
Q13263_TIF1B_HUMAN	TRIM28	7	ND in control
B7ZW38_HNRC3_HUMAN	HNRNPCL3	7	7,00
P62263_RS14_HUMAN	RPS14	7	7,00
P0CW22_RS17L_HUMAN	RPS17L	7	3,50
P62081_RS7_HUMAN	RPS7	7	3,50
Q99459_CDC5L_HUMAN	CDC5L	6	ND in control
Q04637_IF4G1_HUMAN	EIF4G1	6	ND in control
Q12926_ELAV2_HUMAN	ELAVL2	6	ND in control
Q92615_LAR4B_HUMAN	LARP4B	6	ND in control
Q4G0J3_LARP7_HUMAN	LARP7	6	ND in control
P43243_MATR3_HUMAN	MATR3	6	ND in control
P26599_PTBP1_HUMAN	PTBP1	6	ND in control
Q00577_PURA_HUMAN	PURA	6	ND in control
Q9Y2P8_RCL1_HUMAN	RCL1	6	ND in control
P26373_RL13_HUMAN	RPL13	6	ND in control
P62280_RS11_HUMAN	RPS11	6	ND in control
P62277_RS13_HUMAN	RPS13	6	ND in control
Q08945_SSRP1_HUMAN	SSRP1	6	ND in control
O43318_M3K7_HUMAN	MAP3K7	6	6,00
P09874_PARP1_HUMAN	PARP1	6	6,00
P18621_RL17_HUMAN	RPL17	6	6,00
P23588_IF4B_HUMAN	EIF4B	6	2,00
Q8WWY3_PRP31_HUMAN	PRPF31	6	2,00
P62847_RS24_HUMAN	RPS24	6	2,00
Q9NVI7_ATD3A_HUMAN	ATAD3A	5	ND in control
Q8N163_CCAR2_HUMAN	CCAR2	5	ND in control
Q8IWX8_CHERP_HUMAN	CHERP	5	ND in control
P53621_COPA_HUMAN	COPA	5	ND in control
Q15029_U5S1_HUMAN	EFTUD2	5	ND in control
P60228_EIF3E_HUMAN	EIF3E	5	ND in control
O00303_EIF3F_HUMAN	EIF3F	5	ND in control
Q7L2H7_EIF3M_HUMAN	EIF3M	5	ND in control
Q96I24_FUBP3_HUMAN	FUBP3	5	ND in control
O75569_PRKRA_HUMAN	PRKRA	5	ND in control
Q96I25_SPF45_HUMAN	RBM17	5	ND in control

Reference	Gene Symbol	Number of peptides	Fold Change
P62906_RL10A_HUMAN	RPL10A	5	ND in control
Q9UNX3_RL26L_HUMAN	RPL26L1	5	ND in control
P50454_SERPH_HUMAN	SERPINH1	5	ND in control
P38159_RBMX_HUMAN	RBMX	5	5,00
Q07020_RL18_HUMAN	RPL18	5	5,00
P13010_XRCC5_HUMAN	XRCC5	5	5,00
Q14444_CAPR1_HUMAN	CAPRIN1	5	2,50
P17844_DDX5_HUMAN	DDX5	5	2,50
P62244_RS15A_HUMAN	RPS15A	5	2,50
P62753_RS6_HUMAN	RPS6	5	2,50
O15234_CASC3_HUMAN	CASC3	4	ND in control
Q96CT7_CC124_HUMAN	CCDC124	4	ND in control
P12532_KCRU_HUMAN	CKMT1A	4	ND in control
Q7Z478_DHX29_HUMAN	DHX29	4	ND in control
Q6P158_DHX57_HUMAN	DHX57	4	ND in control
Q96N67_DOCK7_HUMAN	DOCK7	4	ND in control
Q13283_G3BP1_HUMAN	G3BP1	4	ND in control
Q92945_FUBP2_HUMAN	KHSRP	4	ND in control
Q9UN81_LORF1_HUMAN	L1RE1	4	ND in control
Q09161_NCBP1_HUMAN	NCBP1	4	ND in control
P06748_NPM_HUMAN	NPM1	4	ND in control
Q9UMS4_PRP19_HUMAN	PRPF19	4	ND in control
Q9UKM9_RALY_HUMAN	RALY	4	ND in control
Q9NW13_RBM28_HUMAN	RBM28	4	ND in control
P46778_RL21_HUMAN	RPL21	4	ND in control
P62888_RL30_HUMAN	RPL30	4	ND in control
P63173_RL38_HUMAN	RPL38	4	ND in control
Q8NHW5_RLA0L_HUMAN	RPLP0P6	4	ND in control
P05387_RLA2_HUMAN	RPLP2	4	ND in control
O00442_RTCA_HUMAN	RTCA	4	ND in control
Q15020_SART3_HUMAN	SART3	4	ND in control
P62316_SMD2_HUMAN	SNRPD2	4	ND in control
Q2NL82_TSR1_HUMAN	TSR1	4	ND in control
Q9Y4E8_UBP15_HUMAN	USP15	4	ND in control
P16989_YBOX3_HUMAN	YBX3	4	ND in control
P62736_ACTA_HUMAN	ACTA2	4	4,00
P33778_H2B1B_HUMAN	HIST1H2BB	4	4,00
P12268_IMDH2_HUMAN	IMPDH2	4	4,00
P62913_RL11_HUMAN	RPL11	4	4,00
Q6NVV1_R13P3_HUMAN	RPL13AP3	4	4,00

Reference	Gene Symbol	Number of peptides	Fold Change
P83731_RL24_HUMAN	RPL24	4	4,00
Q14103_HNRPD_HUMAN	HNRNPD	4	2,00
O15397_IPO8_HUMAN	IPO8	4	2,00
Q86V81_THOC4_HUMAN	ALYREF	3	ND in control
Q8IUX4_ABC3F_HUMAN	APOBEC3F	3	ND in control
Q92974_ARHG2_HUMAN	ARHGEF2	3	ND in control
Q8N3C0_ASCC3_HUMAN	ASCC3	3	ND in control
P25311_ZA2G_HUMAN	AZGP1	3	ND in control
Q07021_C1QBP_HUMAN	C1QBP	3	ND in control
Q9BRJ6_CG050_HUMAN	C7orf50	3	ND in control
Q9P1Y5_CAMP3_HUMAN	CAMSAP3	3	ND in control
P04040_CATA_HUMAN	CAT	3	ND in control
Q14008_CKAP5_HUMAN	CKAP5	3	ND in control
P35606_COPB2_HUMAN	COPB2	3	ND in control
Q9UK59_DBR1_HUMAN	DBR1	3	ND in control
P56537_IF6_HUMAN	EIF6	3	ND in control
Q06787_FMR1_HUMAN	FMR1	3	ND in control
Q96AE4_FUBP1_HUMAN	FUBP1	3	ND in control
P04406_G3P_HUMAN	GAPDH	3	ND in control
Q92947_GCDH_HUMAN	GCDH	3	ND in control
Q6Y7W6_PERQ2_HUMAN	GIGYF2	3	ND in control
P52597_HNRPF_HUMAN	HNRNPF	3	ND in control
Q1KMD3_HNRL2_HUMAN	HNRNPUL2	3	ND in control
Q659C4_LAR1B_HUMAN	LARP1B	3	ND in control
Q71RC2_LARP4_HUMAN	LARP4	3	ND in control
Q96A72_MGN2_HUMAN	MAGOHB	3	ND in control
Q96DH6_MSI2H_HUMAN	MSI2	3	ND in control
Q53EL6_PDCD4_HUMAN	PDCD4	3	ND in control
O43175_SERA_HUMAN	PHGDH	3	ND in control
Q06830_PRDX1_HUMAN	PRDX1	3	ND in control
Q8WXF1_PSPC1_HUMAN	PSPC1	3	ND in control
P27635_RL10_HUMAN	RPL10	3	ND in control
P62910_RL32_HUMAN	RPL32	3	ND in control
P62917_RL8_HUMAN	RPL8	3	ND in control
P05388_RLA0_HUMAN	RPLP0	3	ND in control
P62857_RS28_HUMAN	RPS28	3	ND in control
O76021_RL1D1_HUMAN	RSL1D1	3	ND in control
Q8NC51_PAIRB_HUMAN	SERBP1	3	ND in control
Q9Y3B4_SF3B6_HUMAN	SF3B6	3	ND in control
Q15477_SKIV2_HUMAN	SKIV2L	3	ND in control

Reference	Gene Symbol	Number of peptides	Fold Change
P42285_SK2L2_HUMAN	SKIV2L2	3	ND in control
P09661_RU2A_HUMAN	SNRPA1	3	ND in control
P62304_RUXE_HUMAN	SNRPE	3	ND in control
Q04837_SSBP_HUMAN	SSBP1	3	ND in control
Q9Y5B9_SP16H_HUMAN	SUPT16H	3	ND in control
P42166_LAP2A_HUMAN	TMPO	3	ND in control
Q6PGP7_TTC37_HUMAN	TTC37	3	ND in control
Q7Z2W4_ZCCHV_HUMAN	ZC3HAV1	3	ND in control
Q6NZY4_ZCHC8_HUMAN	ZCCHC8	3	ND in control
Q13867_BLMH_HUMAN	BLMH	3	3,00
Q5W0B1_RN219_HUMAN	RNF219	3	3,00
P61353_RL27_HUMAN	RPL27	3	3,00
Q71UM5_RS27L_HUMAN	RPS27L	3	3,00
Q9UKV3_ACINU_HUMAN	ACIN1	2	ND in control
Q9UL18_AGO1_HUMAN	AGO1	2	ND in control
Q9ULX6_AKP8L_HUMAN	AKAP8L	2	ND in control
O75531_BAF_HUMAN	BANF1	2	ND in control
Q9Y3Y2_CHTOP_HUMAN	CHTOP	2	ND in control
O75175_CNOT3_HUMAN	CNOT3	2	ND in control
Q9UNQ2_DIM1_HUMAN	DIMT1	2	ND in control
Q99848_EBP2_HUMAN	EBNA1BP2	2	ND in control
P13639_EF2_HUMAN	EEF2	2	ND in control
O75822_EIF3J_HUMAN	EIF3J	2	ND in control
P78344_IF4G2_HUMAN	EIF4G2	2	ND in control
Q9NX05_F120C_HUMAN	FAM120C	2	ND in control
P16383_GCFC2_HUMAN	GCFC2	2	ND in control
Q8N954_GPT11_HUMAN	GPATCH11	2	ND in control
Q9BQ67_GRWD1_HUMAN	GRWD1	2	ND in control
Q92522_H1X_HUMAN	H1FX	2	ND in control
P42357_HUTH_HUMAN	HAL	2	ND in control
P16403_H12_HUMAN	HIST1H1C	2	ND in control
B4DY08_B4DY08_HUMAN	HNRNPC	2	ND in control
O14654_IRS4_HUMAN	IRS4	2	ND in control
P33993_MCM7_HUMAN	MCM7	2	ND in control
P82650_RT22_HUMAN	MRPS22	2	ND in control
Q9NX24_NHP2_HUMAN	NHP2	2	ND in control
O15226_NKRF_HUMAN	NKRF	2	ND in control
Q86U42_PABP2_HUMAN	PABPN1	2	ND in control
Q16875_F263_HUMAN	PFKFB3	2	ND in control
P14618_KPYM_HUMAN	PKM	2	ND in control

Reference	Gene Symbol	Number of peptides	Fold Change
Q9H307_PININ_HUMAN	PNN	2	ND in control
Q99575_POP1_HUMAN	POP1	2	ND in control
O60256_KPRB_HUMAN	PRPSAP2	2	ND in control
O43242_PSMD3_HUMAN	PSMD3	2	ND in control
Q96QR8_PURB_HUMAN	PURB	2	ND in control
Q96PU8_QKI_HUMAN	QKI	2	ND in control
P42696_RBM34_HUMAN	RBM34	2	ND in control
Q9Y5S9_RBM8A_HUMAN	RBM8A	2	ND in control
P35251_RFC1_HUMAN	RFC1	2	ND in control
P35244_RFA3_HUMAN	RPA3	2	ND in control
Q02543_RL18A_HUMAN	RPL18A	2	ND in control
P84098_RL19_HUMAN	RPL19	2	ND in control
P46779_RL28_HUMAN	RPL28	2	ND in control
P47914_RL29_HUMAN	RPL29	2	ND in control
P49207_RL34_HUMAN	RPL34	2	ND in control
P42766_RL35_HUMAN	RPL35	2	ND in control
P18077_RL35A_HUMAN	RPL35A	2	ND in control
Q9Y3U8_RL36_HUMAN	RPL36	2	ND in control
P62841_RS15_HUMAN	RPS15	2	ND in control
P60866_RS20_HUMAN	RPS20	2	ND in control
P63220_RS21_HUMAN	RPS21	2	ND in control
Q9Y265_RUVB1_HUMAN	RUVBL1	2	ND in control
Q15459_SF3A1_HUMAN	SF3A1	2	ND in control
Q15427_SF3B4_HUMAN	SF3B4	2	ND in control
P08621_RU17_HUMAN	SNRNP70	2	ND in control
Q13573_SNW1_HUMAN	SNW1	2	ND in control
P84103_SRSF3_HUMAN	SRSF3	2	ND in control
Q8IX01_SUGP2_HUMAN	SUGP2	2	ND in control
O00267_SPT5H_HUMAN	SUPT5H	2	ND in control
Q13148_TADBP_HUMAN	TARDBP	2	ND in control
Q13595_TRA2A_HUMAN	TRA2A	2	ND in control
P62995_TRA2B_HUMAN	TRA2B	2	ND in control
Q8IZH2_XRN1_HUMAN	XRN1	2	ND in control
Q9H6S0_YTDC2_HUMAN	YTHDC2	2	ND in control
Q9BYJ9_YTHD1_HUMAN	YTHDF1	2	ND in control
Q9Y5A9_YTHD2_HUMAN	YTHDF2	2	ND in control
Q96KR1_ZFR_HUMAN	ZFR	2	ND in control
Q9UL40_ZN346_HUMAN	ZNF346	2	ND in control
P62861_RS30_HUMAN	FAU	2	2,00
Q96QV6_H2A1A_HUMAN	HIST1H2AA	2	2,00

Reference	Gene Symbol	Number of peptides	Fold Change
Q13151_ROA0_HUMAN	HNRNPA0	2	2,00
P50914_RL14_HUMAN	RPL14	2	2,00
P46776_RL27A_HUMAN	RPL27A	2	2,00
Q9NQ39_RS10L_HUMAN	RPS10P5	2	2,00
P25398_RS12_HUMAN	RPS12	2	2,00
P46782_RS5_HUMAN	RPS5	2	2,00
P60709_ACTB_HUMAN	ACTB	1	ND in control
Q9UKV8_AGO2_HUMAN	AGO2	1	ND in control
Q9NP73_ALG13_HUMAN	ALG13	1	ND in control
Q9NRW3_ABC3C_HUMAN	APOBEC3C	1	ND in control
P25705_ATPA_HUMAN	ATP5A1	1	ND in control
O75934_SPF27_HUMAN	BCAS2	1	ND in control
E9PRG8_CK098_HUMAN	C11orf98	1	ND in control
Q9H7E9_CH033_HUMAN	C8orf33	1	ND in control
Q96PX6_CC85A_HUMAN	CCDC85A	1	ND in control
P50991_TCPD_HUMAN	CCT4	1	ND in control
Q8N8E3_CE112_HUMAN	CEP112	1	ND in control
Q9NZN8_CNOT2_HUMAN	CNOT2	1	ND in control
Q9NQ92_COPRS_HUMAN	COPRS	1	ND in control
Q5TZA2_CROCC_HUMAN	CROCC	1	ND in control
Q9BQ39_DDX50_HUMAN	DDX50	1	ND in control
Q9BTZ2_DHRS4_HUMAN	DHRS4	1	ND in control
Q8WXX5_DNJC9_HUMAN	DNAJC9	1	ND in control
P24534_EF1B_HUMAN	EEF1B2	1	ND in control
P19525_E2AK2_HUMAN	EIF2AK2	1	ND in control
C9JUF0_C9JUF0_HUMAN	EIF4A2	1	ND in control
P49327_FAS_HUMAN	FASN	1	ND in control
Q9NY12_GAR1_HUMAN	GAR1	1	ND in control
Q08378_GOGA3_HUMAN	GOLGA3	1	ND in control
Q02539_H11_HUMAN	HIST1H1A	1	ND in control
P62807_H2B1C_HUMAN	HIST1H2BC	1	ND in control
Q16695_H31T_HUMAN	HIST3H3	1	ND in control
P51659_DHB4_HUMAN	HSD17B4	1	ND in control
P08238_HS90B_HUMAN	HSP90AB1	1	ND in control
Q58FF8_H90B2_HUMAN	HSP90AB2P	1	ND in control
O00458_IFRD1_HUMAN	IFRD1	1	ND in control
Q9ULR0_ISY1_HUMAN	ISY1	1	ND in control
Q07666_KHDR1_HUMAN	KHDRBS1	1	ND in control
O00505_IMA4_HUMAN	KPNA3	1	ND in control
Q6ZN17_LN28B_HUMAN	LIN28B	1	ND in control

Reference	Gene Symbol	Number of peptides	Fold Change
P42704_LPPRC_HUMAN	LRPPRC	1	ND in control
Q3MHD2_LSM12_HUMAN	LSM12	1	ND in control
Q9Y383_LC7L2_HUMAN	LUC7L2	1	ND in control
Q3KQU3_MA7D1_HUMAN	MAP7D1	1	ND in control
P49736_MCM2_HUMAN	MCM2	1	ND in control
Q9NWU5_RM22_HUMAN	MRPL22	1	ND in control
Q9BYD3_RM04_HUMAN	MRPL4	1	ND in control
Q9Y3D9_RT23_HUMAN	MRPS23	1	ND in control
Q92665_RT31_HUMAN	MRPS31	1	ND in control
O43347_MSI1H_HUMAN	MSI1	1	ND in control
P13995_MTDC_HUMAN	MTHFD2	1	ND in control
P55209_NP1L1_HUMAN	NAP1L1	1	ND in control
O00567_NOP56_HUMAN	NOP56	1	ND in control
O15294_OGT1_HUMAN	OGT	1	ND in control
Q01804_OTUD4_HUMAN	OTUD4	1	ND in control
P0CB38_PAB4L_HUMAN	PABPC4L	1	ND in control
Q58A45_PAN3_HUMAN	PAN3	1	ND in control
Q15365_PCBP1_HUMAN	PCBP1	1	ND in control
Q15366_PCBP2_HUMAN	PCBP2	1	ND in control
Q15084_PDIA6_HUMAN	PDIA6	1	ND in control
Q96HS1_PGAM5_HUMAN	PGAM5	1	ND in control
Q02809_PLOD1_HUMAN	PLOD1	1	ND in control
O60437_PEPL_HUMAN	PPL	1	ND in control
P32119_PRDX2_HUMAN	PRDX2	1	ND in control
O43172_PRP4_HUMAN	PRPF4	1	ND in control
P60891_PRPS1_HUMAN	PRPS1	1	ND in control
P11908_PRPS2_HUMAN	PRPS2	1	ND in control
Q14558_KPRA_HUMAN	PRPSAP1	1	ND in control
P25789_PSA4_HUMAN	PSMA4	1	ND in control
O14818_PSA7_HUMAN	PSMA7	1	ND in control
P20618_PSB1_HUMAN	PSMB1	1	ND in control
Q14671_PUM1_HUMAN	PUM1	1	ND in control
P98179_RBM3_HUMAN	RBM3	1	ND in control
Q14498_RBM39_HUMAN	RBM39	1	ND in control
P29558_RBMS1_HUMAN	RBMS1	1	ND in control
Q15434_RBMS2_HUMAN	RBMS2	1	ND in control
Q15287_RNPS1_HUMAN	RNPS1	1	ND in control
Q96L21_RL10L_HUMAN	RPL10L	1	ND in control
P61513_RL37A_HUMAN	RPL37A	1	ND in control
P05386_RLA1_HUMAN	RPLP1	1	ND in control
P04844_RPN2_HUMAN	RPN2	1	ND in control

Reference	Gene Symbol	Number of peptides	Fold Change
Q5JNZ5_RS26L_HUMAN	RPS26P11	1	ND in control
P42677_RS27_HUMAN	RPS27	1	ND in control
P62273_RS29_HUMAN	RPS29	1	ND in control
C9JQR9_C9JQR9_HUMAN	RPSAP58	1	ND in control
Q92600_RCD1_HUMAN	RQCD1	1	ND in control
Q9NVU7_SDA1_HUMAN	SDAD1	1	ND in control
P05141_ADT2_HUMAN	SLC25A5	1	ND in control
Q9BWU0_NADAP_HUMAN	SLC4A1AP	1	ND in control
O75940_SPF30_HUMAN	SMNDC1	1	ND in control
O95721_SNP29_HUMAN	SNAP29	1	ND in control
P09012_SNRPA_HUMAN	SNRPA	1	ND in control
P08579_RU2B_HUMAN	SNRPB2	1	ND in control
P62314_SMD1_HUMAN	SNRPD1	1	ND in control
Q9UHB9_SRP68_HUMAN	SRP68	1	ND in control
Q07955_SRSF1_HUMAN	SRSF1	1	ND in control
O75494_SRS10_HUMAN	SRSF10	1	ND in control
Q13243_SRSF5_HUMAN	SRSF5	1	ND in control
Q16629_SRSF7_HUMAN	SRSF7	1	ND in control
P30872_SSR1_HUMAN	SSTR1	1	ND in control
B7Z645_B7Z645_HUMAN	SYNCRIP	1	ND in control
Q8N5C8_TAB3_HUMAN	TAB3	1	ND in control
Q5QJ74_TBCEL_HUMAN	TBCEL	1	ND in control
O43734_CIKS_HUMAN	TRAF3IP2	1	ND in control
Q9BRZ2_TRI56_HUMAN	TRIM56	1	ND in control
O75962_TRIO_HUMAN	TRIO	1	ND in control
Q7Z2T5_TRM1L_HUMAN	TRMT1L	1	ND in control
Q9BUF5_TBB6_HUMAN	TUBB6	1	ND in control
P26368_U2AF2_HUMAN	U2AF2	1	ND in control
Q5LJB1_Q5LJB1_HUMAN	UCHL5	1	ND in control
Q9BZI7_REN3B_HUMAN	UPF3B	1	ND in control
Q14694_UBP10_HUMAN	USP10	1	ND in control
Q6EMK4_VASN_HUMAN	VASN	1	ND in control
O43709_WBS22_HUMAN	WBSCR22	1	ND in control
Q9HCS7_SYF1_HUMAN	XAB2	1	ND in control
O14980_XPO1_HUMAN	XPO1	1	ND in control
Q96EC8_YIPF6_HUMAN	YIPF6	1	ND in control
Q8N4Q0_ZADH2_HUMAN	ZADH2	1	ND in control
O43167_ZBT24_HUMAN	ZBTB24	1	ND in control
O75152_ZC11A_HUMAN	ZC3H11A	1	ND in control
Q5VYS8_TUT7_HUMAN	ZCCHC6	1	ND in control
Q5BKZ1_ZN326_HUMAN	ZNF326	1	ND in control
